# Skin sensing and wearable technology as tools to measure atopic dermatitis severity

**DOI:** 10.1002/ski2.449

**Published:** 2024-08-15

**Authors:** Yasmin Khan, Alexandar Todorov, Russel Torah, Stephen Beeby, Michael Roger Ardern‐Jones

**Affiliations:** ^1^ Clinical Experimental Sciences Faculty of Medicine University of Southampton Southampton UK; ^2^ Department of Dermatology Southampton General Hospital University Hospitals Southampton NHS Foundation Trust Southampton UK; ^3^ School of Electronics and Computer Science University of Southampton Southampton UK

## Abstract

Wearable medical technology encompasses a range of electronic devices that act as biosensors. Atopic dermatitis (AD) is the commonest inflammatory skin disease and represents an important area of need in which to leverage the power of wearable biosensor technology, especially as the impact of COVID‐19 increases the likelihood of virtual consultations becoming an integrated part of clinical practice. The aim of this review is to systematically define the published evidence for the utility of wearable biosensors in assessment and management of atopic dermatitis (AD). A systematic literature search was conducted for publications from 1995 onwards for ‘sensor’ OR ‘sensing’ OR ‘biosensor’ OR ‘biomarker’. Results were combined (‘AND’) with a search for ‘wearable’ OR ‘actigraphy’ OR ‘Internet of things’ OR ‘microneedle’ OR ‘patch’ OR ‘e‐textile’ OR ‘smart textile’ and atopic dermatitis (MESH terms). Fifty seven abstracts were identified from the database search of which 39 were selected for detailed review. Broadly, wearable sensing systems in atopic dermatitis were split into three categories: wearable biosensor modules (actigraphy and smartwatches), clothing and integrated fabrics placed onto the epidermis and intradermal or subcutaneous sensors. The best evidence for correlation with AD disease severity was with actigraphy measurements of itch. However, newer approaches including sensing skin barrier function, inflammation and small molecule analysis as well as employing artificial intelligence offer more potential for advanced disease monitoring. Skin diseases, specifically AD, stand to benefit greatly from wearable technology, because of the ease of direct contact to the skin, the high prevalence of the disease and the large unmet need for better disease control in this group. However, important emphasis must be placed on validating the correlation of data from such technology with patient‐reported outcomes. Wearable biosensors offer a huge potential to deliver better diagnostics, monitoring and treatment outcomes for patients.

## Graphical abstract

This article explores the role of wearable biosensor technology in managing atopic dermatitis (AD), emphasising the potential to enhance disease monitoring and treatment outcomes through devices that measure parameters such as itch, skin barrier function and inflammation. The systematic review highlights various wearable technologies including wrist actigraphy, smartwatches and integrated fabrics and assesses their effectiveness based on existing clinical evidence. The findings suggest that wearable biosensors could significantly improve the objective assessment of AD, facilitating more personalised and responsive treatment strategies in both clinical and remote settings.

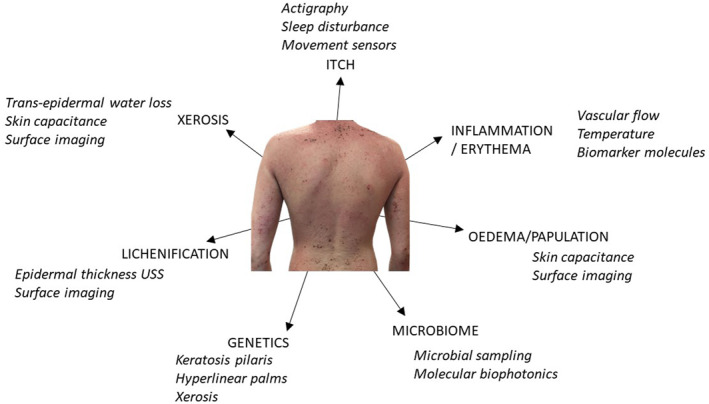

**What is already known about this topic?**

- Wearable biosensors have been utilised in various medical fields for monitoring physiological parameters, but their application in dermatology, particularly for atopic dermatitis (AD), has been less explored.
- The power of recent technological advancements to detect fine differences in materials can be applied to dermatology.
- For assessment of skin disease, skin sensing and wearable technologies are increasingly available.

**What does this study add?**

- The review compiles evidence on wearable biosensors for atopic dermatitis, showcasing their enhanced precision in monitoring symptoms like itch and skin barrier functions.
- It highlights the suitability of skin‐sensing technologies for crucial measurements of skin barrier function essential in atopic dermatitis management.
- The review points to the ongoing development of devices for assessing scratching, barrier function, inflammation and molecules, underlining wearable technology's future role in healthcare and remote assessments.

## 1 INTRODUCTION

Medical devices are products or equipment intended for primarily medical use and encompass a range of electronic devices that can be placed on or in the body.^1^ Sensors including measurement of blood oxygen saturations, electrocardiography and blood pressure have been employed for many years in hospitals. Until recently, outpatient or community medical sensors have mostly been implantable devices, such as pacemakers. However, there is increasing interest in the use of non‐implantable, or wearable devices for medical applications (wearable biosensors). These can be incorporated into clothing, adherent to the skin, penetrate the epidermis, or inserted into a body orifice. Medical devices commonly serve to monitor and record an individual's physical and/or biochemical parameters prior to computing, relaying or actioning the data as required.^2^

Dermatology is uniquely placed to benefit from such wearable biosensors because of the ease of access to the skin and the importance of dynamic changes in cutaneous features in the diagnosis and management of disease. Atopic dermatitis (AD) is the commonest inflammatory skin disease and represents an important area of need in which to leverage the power of wearable biosensor technology. Currently for AD, in the clinic, diagnosis and measurement of disease activity relies on a dermatologist's visual assessment and subjective patient‐reported metrics such as severity of itching and impact on quality of life. However, even the best measures used by dermatologists for assessing disease severity such as the Eczema Area and Severity Index score (EASI) are known to show only a moderate interobserver reliability.^3^ Furthermore, AD is a condition which changes daily in severity, but while repeated measures over days or weeks would be more reliable, they are not practical for current clinical use. Therefore, the lack of an objective metric, with which to track a patient's AD severity and response to treatment, can make clinical management challenging and be frustrating for patients.^4^

**FIGURE 1:**
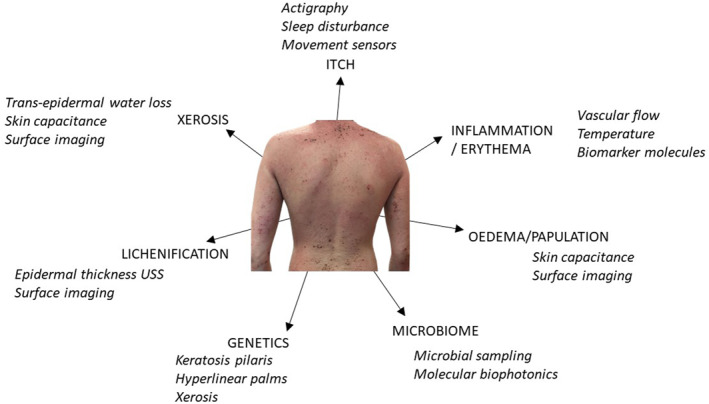
A figure to show the key clinical features of atopic dermatitis and to illustrate examples of devices that are available or could be incorporated into wearable technologies to measure disease severity.

**FIGURE 2:**
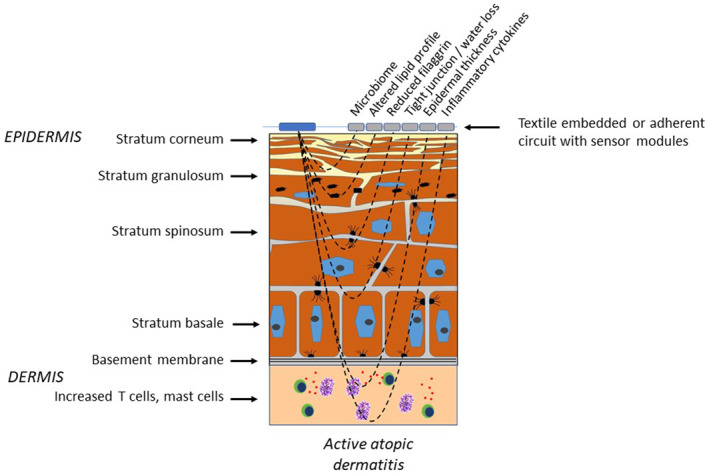
An illustration to show how key pathways in atopic dermatitis pathogenesis could be detected using skin‐sensing technology.

Indeed, the clinical features of AD lend themselves to assessment with wearable technology (Figure 1). However, in addition, measurements of key pathological pathways may offer more sensitive and more specific measures of response to treatment (or lack of) at early time points in response to treatment (Figure 2). The growing expectancy that virtual consultations will form part of modern clinical practice adds to the need for reliable systems to interrogate disease severity in a virtual environment. We set out to systematically define the published evidence for the utility of wearable biosensors in assessment and management of atopic dermatitis.

## 2 METHODS

Systematic review of the bibliographic databases (PubMed, Medline and Google scholar) was performed in accordance with the Preferred Reporting Items for Systematic Reviews and Meta‐Analyses (PRISMA) statement. In brief, publications from 1995 to July 2024 were searched for ‘sensor’ OR ‘sensing’ OR ‘biosensor’ OR ‘biomarker’. Results were combined (‘AND’) with a search for ‘wearable’ OR ‘actigraphy’ OR ‘Internet of things’ OR ‘microneedle’ OR ‘patch’ OR ‘e‐textile’ OR ‘smart textile’ and atopic dermatitis (MESH terms). Only articles written in English were included with no restriction on the type of article.

## 3 RESULTS

Fifty seven abstracts were identified from the database search. 18 papers were excluded as not relevant, 39 were selected for detailed review.

### 3.1 Wearable biosensor modules

Wearable biosensor modules are a category of electronic devices, which are integrated into objects that can be worn by an individual. Examples include devices within wristbands, wrist watches and patches.^5^ The key developments are summarised in Table 1.

**TABLE 1:** Comparison of wearable biosensor technologies for atopic dermatitis.

Sensor type	Application	Measurement accuracy	Limitations	Development stage	Conclusion
Wrist actigraphy	Measurement of itch	High accuracy for night‐time scratching	Limited to nocturnal use; does not detect other arm's movements	Widely used in clinical trials	High effectiveness for measuring scratching and sleep disturbances
Smartwatches	Monitoring scratching behaviour	High accuracy (>90% for night‐time scratching)	Daytime movements interfere with measurements	Under development for enhanced capabilities	Positive feedback; increased confidence in treatment management
Integrated fabrics	Skin properties (hydration, temperature)	Varies; generally high for specific parameters	Limited by flexibility and sensor integration with textiles	Experimental, some commercial applications	Promising for continuous monitoring of skin health
Microneedle sensors	Long‐term biomarker monitoring	High accuracy in stable environments	Potential issues with biofouling; limited long‐term data in dermatology	Mainly used in research settings	Effective in theory, needs more data for AD applications

#### 3.1.1 Wrist actigraphy

Pruritus (or itch) is the principal symptom of atopic dermatitis^6^ and is the dominant driver for loss of quality of life in AD. Itch is often defined as an uncomfortable sensation of the skin which causes a desire to scratch and thus the physical act of scratching has been used in attempts to objectively quantify itching. Since the early 2000s, researchers have attempted to measure scratching. The technologies have included watches containing accelerometers, measurement of forearm muscle potentials, piezoelectric devices applied to the fingernails, sound recording devices and electromagnetic movement detection.^7^ Actigraphy has been one of the more successful methods to record scratching behaviour and recent advances in the technology show it to be precise, accurate and reliable in clinical use.^8^ A wrist actigraph is a portable device which uses an accelerometer to measure wrist movement as a proxy for scratch.^8^ Pruritus measurements from a wrist actigraph have been validated against infrared video recordings.^7^ A non‐intrusive DigiTrac monitor placed on the wrist has also been shown to correlate closely with objective clinical SCORAD (Scoring Atopic Dermatitis) data and peripheral blood AD chemokine markers. The authors recommended that wrist activity between 1 and 3 Hz in the first 3 hours of sleep was an insightful indicator of AD severity in children.^9^ Actigraphy can also provide data on sleep disturbance in patients with AD.^10^ Actigraphy has also been reported to be measurable using a wireless sensor patch applied directly to the skin of the dorsal hand.^11^ The main limitation to accuracy in actigraphic measurement is differentiation between scratch movement and non‐scratch movements. To address this, some groups have employed machine learning algorithms to enhance accuracy,^12^, ^13^ but despite these improvements, the technology only achieves a moderate ability to correctly identify scratching events and to capture all scratching events (*F*1 = 0.45).^13^

#### 3.1.2 Smartwatches

A smartwatch is a device that acts both as a time‐keeping device and a ‘general purpose networked computer with an array of sensors’.^14^ A three‐phase study was carried out with 40 participants and two dermatologists in order to assess a smartwatch‐based mobile device to monitor scratching behaviour.^4^ Both dermatologists and patients found the device useful for AD management and increased confidence in the measurement of treatment response. Further iterations of the smartwatch approach confirmed that it was able to identify night‐time scratching behaviour with >90% accuracy.^15^ The limitations of a smartwatch‐based design to measure itch include the fact it can only be used to measure nocturnal symptoms due to the confounding effects of daytime movements and the lack of detection of scratching by the other arm, which is especially relevant if eczema is present on the arm of the watch.^4^ Furthermore, these devices do not have any ability to measure the appearance of skin in atopic dermatitis (without user input), which is a key feature of current scoring systems used for AD severity.

Beyond atopic dermatitis, smartwatches have rapidly penetrated the healthcare space since 2014 and are projected to have the largest share of growth in the wearable medical devices market.^16^ A systematic review^17^ on the use of smartwatches in health and wellness deemed them to have the potential to transform health care by enabling near real‐time monitoring of physiological measures and physical activity, whilst also allowing in situ mini surveys and behaviour verification. Smartwatches have been showed to accurately measure the heart rate over a five‐minute period daily for 30 days with a difference of 0.89 bpm when validated against an established chest Bio‐harness.^18^ AD is a stress‐responsive disorder that involves autonomic nervous system dysfunction and thus heart rate variability could indicate itching or distress in patients with AD offering another avenue for monitoring.^19^ Smartwatches are also already able to accurately track step count and physical activity^20^ which may offer insight into the activity of patients with AD which may be linked to their quality of life. Increasingly smartwatch biosensors are being developed to monitor skin temperature,^18^ light intensity exposure,^21^ eating behaviour,^22^ oxygen saturation and respiratory rate.^16^

In addition to automated input of biosensing data, wearable accessories such as smartwatches offer the potential to record user input and responses during daily activities. Mobile wellness loggers employ a self‐monitoring system to manage chronic diseases like diabetes, heart failure and sleep disorders.^4^ They have also been beneficial in depression via high frequency assessment of cognition and mood over an extended period using an Apple Watch in people with major depressive disorder.^23^ In current practice, 65% of AD patients reported being unable to effectively convey the impact of skin disease to their doctor. Utilising smartwatch technology and user self‐reporting an application was developed which promoted the wearer to manually input adjustable parameters such as self‐reported daily mood, foods ingested, intensity and frequency of itch and personal notes,^24^ thus facilitating the monitoring of AD severity, response to treatment and effect on quality of life.

### 3.2 Integrated fabrics, textiles and surface material wearable biosensors

Electronic textiles (e‐textiles), where electronic functionality is incorporated into textile substrates, is a promising field in wearable medical equipment and skin sensing.^25^ In recent years, these systems have been developed to measure a number of skin properties such as hydration, temperature, blood oxygen level, wound healing and skin mechanics.^2^

#### 3.2.1 Clothing

Wearable electronic technology in clothing offers the promise of flexibility, distortion tolerance and permeability which allows such sensors to measure large areas of skin.^26^ These designs also overcome the problem of prolonged contact of a less flexible non‐fabric based sensor with the naturally mobile and soft skin surface.^27^ A fabric temperature sensor, composed of a continuous metal fibre woven into non‐conductive yarn, was able to accurately detect skin temperature (error ±0.2°C) with a short response time.^25^ Another fabric‐based design, the ‘Hexoskin’ wearable vest, was capable of monitoring heart rate and breathing rate during daily activity.^28^ Other textile‐based wearable devices and sensors^29^, ^30^ have been shown to track physical activity, heart rate, ECG rhythms, sweat and sweat rate,^31^ SpO2 and body temperature.^32^

Small molecule measurements have also been shown to be possible in fabric‐based designs. Biochemical sensors such as glucose oxidase‐ and lactate oxidase‐based electrodes have been incorporated into fabrics allowing an accurate measurement of glucose and lactate production. Fabric‐based wearable biosensors offer an exciting possibility for AD because of the potential to measure disease‐relevant physiology such as transepidermal water loss (TEWL), skin temperature^33^ and inflammation over large areas of skin in a non‐hospital setting.

#### 3.2.2 Bandages and masks

Smart bandages with continuous evaluation of temperature and pH sensors have been previously tested to monitor healing of chronic wounds.^34^ This sensor was successfully integrated within a closed‐loop system that released drugs (via a thermally responsive drug carrier incorporated into a hydrogel patch) into the wound when signs of inflammation or infection were detected in real time. Similar materials may be used in the context of AD in assessing and managing severe AD with superimposed infection. 3D mask devices, made of soft silicone, have also been developed to monitor skin moisture levels on the face in real time and provide recommendations for skin care.^35^ Due to the importance of skin barrier hydration status in determining AD severity, ability to monitor moisture may be utilised in AD.

#### 3.2.3 Biosensors applied directly or adherent to the epidermis

To obtain fixed proximity of wearable sensors to the skin, it is necessary to use adherent sensors such as patches or adherent fabrics. In an attempt to develop an objective measurement of AD severity, epidermal probes such as the Tewameter TM210 and Corneometer CM825^36^ have been used to record transepidermal water loss as a measurement of skin barrier function and capacitance of the surface of the skin as a measurement of stratum corneum hydration. Using such non‐invasive approaches to measure barrier function, the Objective Severity Assessment of Atopic Dermatitis (OSAAD) score^36^ has been developed and shown to correlate with the SCORAD index used in clinical practice. Although this approach was not directly wearable, engineering approaches to minimise the TEWL and capacitance measurements as applied skin sensors is underway.

A soft, battery‐free, reusable skin hydration sensor adherable to the body surface, which wirelessly transmitted the information to a compatible smartphone^37^ was used in pilot studies in AD, psoriasis, urticaria and rosacea. The skin hydration sensor allowed the detection and mapping of skin disease, discerning AD lesions (*p* = 0.0034) and psoriasis lesions (*p* = 0.0156) and detecting trends in dermal diseases like urticaria and rosacea following treatment. A similar patch‐like sensor was developed by Shi et al.^38^ to measure exposure to UV radiation in order to try to prevent the long‐ and short‐term cutaneous complications of excess UV light. The ultra‐thin breathable sensor accurately quantified solar radiation and contained dyes that changed colour when the threshold of exposure was reached. A conforming pressure sensor made from ultra‐thin piezoelectric and semiconductor materials on elastomer substrates facilitates high‐sensitivity measurements of changes in skin turgor, and when applied to multiple sites (neck and arm) was shown to be able to measure blood pressure.^39^ Assessment of skin moisture and skin turgor may be used in the treatment of AD. In addition, measurement of sweat composition in wireless patches as a ‘lab on a chip’ approach are under development.^40^, ^41^ Patches have also been developed to measure movement (and therefore scratching) which can be linked wirelessly to a recording device and because of this are more suitable for long‐term measurements and also for children.^11^

### 3.3 Sensors inserted into the epidermis or dermis

#### 3.3.1 Transdermal sensors

AD represents a chronic inflammatory state in the skin, which is mediated by soluble mediators such as cytokines and chemokines. Furthermore, inflammatory processes modify redox potentials within cells and outside. As a result of the localised nature of AD, such signals are hard to detect in the blood/plasma and therefore direct cutaneous sampling has been explored.^42^ Transdermal monitoring most often targets the interstitial fluid (ISF) residing in the dermis, although peripheral blood may be sampled from deeper layers of the skin.^43^ ISF, which surrounds the parenchymal cells, blood and lymphatic vessels, is formed via extravasation of plasma from capillaries. ISF contains a combination of serum and cellular materials and plays an important role in the transport of signalling molecules between cells, in carrying antigens and cytokines to local draining lymph nodes, and in the transport of nutrients and waste—it is thus a valuable source of biomarkers. With the development of microneedles, there has been a surge in studies to assess their use in biosensing and extraction of biological fluid.^44^

### 3.4 Transdermal sensing of ISF can broadly be split into two categories

(i) ISF harvested via microfluidic extraction for offline analysis
(ii) Microneedle sampling of ISF with biosensors inserted directly into the skin.^43^

ISF sampling via hollow microneedles to an adjacent chamber containing the electrochemical biosensor can be used for monitoring purposes such as glucose levels in diabetics.^45^ A similar device has been used to measure potassium levels.^46^ An interesting refinement is to integrate the sensor into the microneedle. As a proof of concept to test microneedle measurement of drug‐levels, this has been trialled with vancomycin monitoring where the inner lumen of a microneedle contained a peptide with an affinity for vancomycin.^46^ Microfluidic devices extracting ISF are limited in their ability for continuous monitoring by flow of ISF and disposal of analyte. To some degree, improved flow can be achieved by using a dialysis approach.^47^

Antibody‐modified microneedles can allow specific binding of a biomarker of interest in the ISF for subsequent laboratory‐based assays.^48^ For example, minimally invasive microneedles have also been proposed for melanoma screening. The electrochemical device is capable of detecting the expression of tyrosinase,^49^ which is a cancer biomarker involved in the synthesis of melanin, and high levels are found in cutaneous melanoma. Antibody‐coated microneedles may also be valuable for point of care testing.^50^ AD is characterised by immune‐mediated inflammation and epidermal barrier dysfunction. Measurement of key cytokines, including IL‐4 and IL‐13 with biodegradable hyaluronic acid‐loaded microneedle patches, has been shown to be possible in lesions, and changes in measured levels broadly correlate with clinical improvement.^51^ Thus, microneedle sampling of ISF and detection of inflammatory cytokines offers another avenue in AD monitoring.

#### 3.4.1 Subcutaneous sensors

Subcutaneous‐implanted biosensors can provide accurate and reliable information whilst fully integrated in the local environment. They have the potential for sustained accurate monitoring of biomarkers in subcutaneous fluids, thus delivering valuable information about the individual's health, important in chronic disease.^43^ The majority of advancements in subcutaneous sensing applies to glucose monitoring in diabetes. There are several FDA‐approved commercially available implantable glucose sensors, the majority of which are based on electrochemical enzymatic sensing.^52^ Problems reported with these sensors include a short lifetime and sensor calibration issues.^53^ Another problem for implanted sensors is non‐specific protein absorption when interfaces interact with complex biological media. Such ‘biofouling’ can lead to decreased sensor performance.^54^ There is limited data available on subcutaneous sensors in skin disease.

### 3.5 Future developments

Research in flexible and stretchable electronics has demonstrated future platforms for non‐invasive medical sensing that will provide an interesting option for skin monitoring applications and we have previously reviewed the potential of radiofrequency reflectometry for this purpose.^55^ A further prominent example is work utilising nanotechnology to develop temporary tattoos capable of use as skin‐sensing electrodes.^56^, ^57^ These devices are highly flexible, exhibit excellent resistance to mechanical stress and allow for longer sensing operation due to their unobtrusive nature.^57^ The devices can achieve real‐time electrochemical analysis of important electrolytes and metabolites, for example, monitoring varying levels of glucose in human sweat.^58^ According to Ono et al., patients with AD have increased glucose levels in their sweat, thereby monitoring this expression can give insight into the condition's severity.^59^ The tattoo sensors can also be used to perform various electrogram procedures and can detect skin temperature and hydration, as shown by Ameri et al.’s Graphene Electronic Tattoo, which can measure electrocardiogram (ECG) and quantify skin hydration while having a thickness of less than 500 nm.^60^ This paper has also highlighted the importance of dermal hydration level in the diagnosis of AD. Tattoo‐based skin sensors can monitor these parameters whilst allowing the user to continue daily routines, but energy management and data processing remain key research challenges due to current technical limitations regarding the integration of power and digital processing systems into an ultra‐thin skin‐like package. We have previously shown that some of these challenges can be overcome including weaving sensors into textiles such as accelerometers^61^ and energy harvesting from nanogenerators within a woven structure.^62^

Regarding current platform technologies, only e‐textiles can accommodate fully independent AD monitoring systems with all necessary components for data collection, analysis and power management into a single garment‐based device. Recent advances in textile‐based optical photoplethysmography (PPG) can be used to monitor parameters associated with AD. Ballaji et al. present a textile sleeve for monitoring oxygen concentration in blood vessels beneath the skin using PPG.^63^ The device utilises light‐emitting and light‐monitoring components and by varying the operating wavelength of the sensor, this method can be modified to measure parameters at a shallower depth in the skin—thereby targeting water concentration within the epidermis and stratum corneum. This has been demonstrated using a method called diffuse reflectance spectroscopy using a standard spectrophotometer and rigid bulky components, but this has yet to be replicated within a wearable e‐textile AD monitoring device.^64^ A standalone device that can replicate the process of diffuse reflectance spectroscopy in a small flexible package like a textile patch shows good promise. Other approaches to measuring core outcomes for AD are also under development, including touch‐free measurement of scratching by analysis of radio wave deflections within a confined space to compute movements of individuals within it, which has been of interest to the industry.^65^, ^66^

### 3.6 Challenges and barriers to implementation

Wearable skin sensors face significant challenges that limit their widespread adoption in clinical practice. Primarily, this is because of their key utility: the potential to offer continuous health monitoring. This primary aim requires the technological adaptation of devices to be both highly functional and user‐friendly. They need to record accurate biosignals over long periods without clinician or technician intervention, demanding advanced materials for skin conformability and biocompatibility. The materials used must effectively interface with skin without causing discomfort yet adhere closely to skin or penetrate very small amounts, without causing allergic contact dermatitis reactions, particularly relevant for individuals with atopic dermatitis. Consequent to the natural process of the skin to sweat, the design complexities increase with the need for these devices to be breathable, allowing for air and moisture passage to maintain skin integrity during prolonged use. Failure to do so results in a gradual increase in sensor humidity and sweat electrolytes that can interfere with primary skin sensing. Although many current materials often struggle to balance flexibility, durability and the necessary exchange properties, complicating their practical application in diverse clinical environments, newer technologies such as the use of breathable clothing designs, temporary tattoos or those that adjust for sweat‐related changes offer hope that this issue will be overcome.

Size and miniaturisation is also a key challenge. Consideration of the benefit of integration of multiple functionalities within a single wearable device is likely to be advantageous, and the most effective sensors may require the coexistence of biophysical and biochemical sensors in a compact form factor that can manage data collection, processing and transmission seamlessly. Such sensors must avoid interference and maintain accuracy across various physiological measurements, which requires sophisticated engineering to mitigate crosstalk and ensure reliable sensor performance.

Finally, powering these multifunctional devices is also a critical development issue. Traditional battery solutions may be too bulky or heavy, detracting from the wearability aspect. Innovative solutions like energy harvesting and wireless power transfer are being explored, but these technologies are still in developmental stages and pose their own set of challenges regarding efficiency and consistent performance in real‐world conditions. These technological and practical barriers must be addressed to facilitate the clinical adoption of wearable skin sensors, improving patient outcomes through better monitoring and intervention strategies.

Before widespread uptake into clinical practice, it is necessary to further validate sensor readouts against clinical disease. In the context of atopic dermatitis, this is likely in the first phase to require cross‐validation against current clinical ‘gold standards’ such as correlation with EASI (or regional EASI), but subsequently may help develop best practice management. For example, the measurement of blood pressure, while frequently undertaken in the clinician's office, is often unreliable, and instead it has become mainstream to offer ambulatory blood pressure monitoring. In the same way, when patients with AD are reviewed in the clinic, single assessment is not a reliable measure of disease activity. Instead, continuous monitoring as offered by sensor technology may prove better. However, qualitative analyses to cross correlate sensor readouts with patient‐reported outcomes is critical to patient‐centred care. Subsequent clinical trials will also be required to demonstrate the benefit to patients of sensing devices in their role to optimise the treatment pathway for atopic dermatitis.

## 4 CONCLUSIONS

Wearable sensors have been widely adopted into society for personal health monitoring and their use is continuing to increase year on year.^16^ As healthcare shifts to an increasingly personalised medicine approach, the facility to offer out of hospital monitoring will further drive the expansion in application and utility of wearable biosensors. In this way, it is critical to retain the patient at the centre of the aims for further development and the ambition to employ skin sensor technology in clinical practice. Therefore, cross validating the utility of such advances with benefits in terms of patient‐reported outcome measures is an important requirement.

Skin diseases, especially AD, stand to benefit greatly from such advances in wearable technology because of the large unmet need in a better system to accurately monitor disease flares and response to treatment. Devices to measure pruritus have been shown to correlate with measures of disease severity in AD, and therefore offer potential for uptake into clinical practice but are limited to night use and are less ideal for arm disease. However, more advanced systems, which include capabilities to sense barrier function, inflammation and even small molecule analysis are under development and offer the exciting possibility of genuine home monitoring. By linking these exciting developments with advances in artificial intelligence to create automatic response systems, these systems will deliver the ultimate goal of real‐time optimization of treatment to transform care for patients with skin disease.

## CONFLICT OF INTEREST STATEMENT

Stephen Beeby and Russel Torah are directors of Smart Fabric Inks Ltd and retain patent WO2020074923A1. Michael Roger Ardern‐Jones (or his department) is a consultant/speaker/grant holder/travel recipient from AbbVie, Almirall, Amgen, Ducentis, Galderma, Janssen, Leo Pharma, Lilly, Novartis, Pfizer, Regeneron, Sanofi Genzyme, UCB, Unilever. Yasmin Khan, Alexandar Todorov declare no conflicts of interest.

## AUTHOR CONTRIBUTIONS

**Yasmin Khan**: Data curation (equal); formal analysis (equal); investigation (equal); methodology (equal); writing – original draft (equal); writing – review & editing (equal). **Alexandar Todorov**: Investigation (equal); writing – review & editing (equal). **Russel Torah**: Data curation (equal); formal analysis (equal); investigation (equal); writing – review & editing (equal). **Stephen Beeby**: Conceptualisation (equal); data curation (equal); formal analysis (equal); supervision (equal); writing – review & editing (equal). **Michael Roger Ardern‐Jones**: Conceptualisation (equal); data curation (equal); formal analysis (equal); investigation (equal); methodology (equal); project administration (equal); resources (equal); software (equal); supervision (equal); writing – review & editing (equal).

## ETHICS STATEMENT

Not applicable.

## PATIENT CONSENT

Written patient consent for publication was obtained.

## Data Availability

Data sharing is not applicable to this article as no datasets were generated or analysed during the current study.
